# Structural Impairments of Hippocampus in Coal Mine Gas Explosion-Related Posttraumatic Stress Disorder

**DOI:** 10.1371/journal.pone.0102042

**Published:** 2014-07-07

**Authors:** Quan Zhang, Chuanjun Zhuo, Xu Lang, Huabing Li, Wen Qin, Chunshui Yu

**Affiliations:** 1 Department of Radiology and Tianjin Key Laboratory of Functional Imaging, Tianjin Medical University General Hospital, Tianjin, China; 2 Department of Psychiatry, Anning Hospital of Tianjin City, Tianjin, China; 3 Department of Radiology, Jinmei Group General Hospital, Jincheng, Shanxi, China; University of Electronic Science and Technology of China, China

## Abstract

Investigations on hippocampal and amygdalar volume have revealed inconsistent results in patients with posttraumatic stress disorder (PTSD). Little is known about the structural covariance alterations between the hippocampus and amygdala in PTSD. In this study, we evaluated the alteration in the hippocampal and amygdalar volume and their structural covariance in the coal mine gas explosion related PTSD. High resolution T1-weighted magnetic resonance imaging (MRI) was performed on coal mine gas explosion related PTSD male patients (n = 14) and non-traumatized coalminers without PTSD (n = 25). The voxel-based morphometry (VBM) method was used to test the inter-group differences in hippocampal and amygdalar volume as well as the inter-group differences in structural covariance between the ipsilateral hippocampus and amygdala. PTSD patients exhibited decreased gray matter volume (GMV) in the bilateral hippocampi compared to controls (*p*<0.05, FDR corrected). GMV covariances between the ipsilateral hippocampus and amygdala were significantly reduced in PTSD patients compared with controls (*p*<0.05, FDR corrected). The coalminers with gas explosion related PTSD had decreased hippocampal volume and structural covariance with the ipsilateral amygdala, suggesting that the structural impairment of the hippocampus may implicate in the pathophysiology of PTSD.

## Introduction

Posttraumatic stress disorder (PTSD) is an anxiety disorder observed in people who have been exposed to severe emotional or physical life-threatening traumatic events (such as war, sexual abuse, and natural disasters), and reacts with intense fear, helplessness or horror. PTSD is characterized by multiple chronic and disabling symptoms, including re-experiencing the event, avoidance of clues related to the trauma, and hyperarousal. It has been proposed that the structural and functional changes of the emotional neurocircuitry, mainly consisted of the amygdala, medial prefrontal cortex and hippocampus, may contribute to the development of PTSD [1].

The relationship between the amygdala and the medial prefrontal cortex was investigated deeply in PTSD by many consistent studies, demonstrating that one candidate neural mechanism of PTSD may be the failure of medial prefrontal cortex in suppressing the hyperresponsivity of the amygdala to threat-related stimuli, which mediates a core symptom of hyperarousal of PTSD [1], [2]. The PTSD is also reported to be closely related to the dysfunction of emotional memory processing. The amygdala plays a critical role in memory of stressful events [3], and the hippocampus is involved in affective processing [4], [5]. The structural alteration of hippocampus has received abroad attention in PTSD. However, the results on gray matter volume (GMV) of the hippocampus in PTSD are controversial. Although many studies reported a reduced hippocampal volume in PTSD patients compared with Non-PTSD subjects [6]–[14], several studies failed to find such GMV differences [15]–[18]. Several meta-analyses confirmed reduced hippocampal volume in PTSD patients; however, the laterality effects are inconsistent. For example, some studies reported GMV reduction in the bilateral hippocampi [6], [19], [20], and others revealed GMV reduction only in the right hippocampus [21], or in the left hippocampus [22], in PTSD patients. The heterogeneous properties of the PTSD subjects, including gender, age, trauma type, duration of trauma exposure, severity of trauma, and elapsed time since trauma [6], [19], [23], may account for the aforementioned inconsistent results regarding hippocampal volume in PTSD. The reports on the structural changes in amygdala in PTSD were fewer and also inconsistent. Increased [24], and decreased [25], [26], amygdala volume was both reported in PTSD, Two meta-analyses both showed on significant alteration of amygdala volumes in PTSD [27], [28].

Relative to the structural alterations of the hippocampus and amygdala, the structural covariance between the two regions has not been reported in PTSD. The interaction between hippocampus and amygdala is important for processing the emotional memory under normal condition [29]–[31]. The amygdala and the hippocampus have real anatomical connectivity [32], [33]. A pilot study revealed that the GMV of the hippocampus covaries strongly with the amygdala in healthy subjects [34]. Structural covariance quantifies the extent to which structure covaries among different brain regions across individuals. Although the biological meaning of this structural covariance remains controversial, it appears to reflect mutually trophic influences or common experience-related plasticity [35], [36], and may reflect synchronized development of covarying brain regions. Covariance methods can be theoretically applied to any structural or anatomical phenotype (such as surface area, GMV, and diffusion indices, etc.) and could provide additional information regarding the functional or structural networks. Networks of structural covariance partially recapitulate the functional networks of healthy subjects and allow the exploration of abnormal structural covariance networks related to various neurological and psychiatric diseases [36]. Therefore, evaluating the alteration of structural covariance between the amygdala and the hippocampus may broaden our insight into the pathogenesis of PTSD.

The structural changes of the hippocampus and amygdala in PTSD patients resulted from combat, natural disaster, child abuse, and civilian assaults were extensively investigated. However, only one research group had reported the hippocampal structural alteration in coal mining disaster-related PTSD. They found that coal mine floods-related PTSD subjects had significantly reduced fractional anisotropy value in bilateral hippocampal body [37], and decreased volume and density in the left anterior hippocampus compared with survivors without PTSD [38]. The victims of the coal mine disaster had high homogeneity in demographic background, trauma intensity, and duration of trauma exposure, which offers an advantage in evaluating the PTSD-related structural brain damage.

In this study, we recruited a group of male coalminers with coal mine gas explosion-induced PTSD and a group of matched non-traumatized coalminers to investigate the alterations in GMV of hippocampal and amygdala, and further investigate alterations in GMV covariance between the hippocampus and the amygdala in PTSD. We hypothesized that the hippocampal volume was reduced, and GMV covariance between hippocampus and the ipsilateral amygdala was disrupted in PTSD patients who have suffered from a coal mine gas explosion.

## Methods

### Subjects

A coal mine gas explosion disaster occurred in February 2006 in Shanxi province of China. Thirty male survivors from the disaster were diagnosed as PTSD according to the Diagnostic and Statistical Manual of Mental Disorders (DSM-IV) criteria at 6 months after trauma. Seven years later, 24 of the 30 victims with PTSD were recruited again for this MRI study. They were re-evaluated by a trained psychiatrist to ensure the persistence of PTSD. Clinician-Administered PTSD Scale (CAPS) was used to assess symptom severity of each patient. Twenty five male coalminers without exposure to the trauma, the colleagues of the victims, were enrolled as controls.

Conventional MR images were used for excluding subjects with visible brain lesions. All controls exhibited a normal MRI appearance. Eight PTSD patients were excluded for visible brain lesions (encephalomalacia, tumors, or cysts). Two patients were further excluded because they met diagnostic criteria only for the lifetime but not for the current PTSD. Only the remaining 14 patients with current and lifetime PTSD were used for further analysis. For each subject, anxiety and depression symptoms were assessed using the Hamilton Rating Scale for Anxiety (HAMA) and Depression (HAMD-24 items version), respectively. The experiment was approved by the Ethical Committee of Tianjin Medical University General Hospital and written informed consent was obtained from each subject before the study.

### MRI acquisition

MR images were obtained on a 3.0 T scanner (Magnetom Verio, Siemens, Erlangen, Germany). A T1-weighted volumetric magnetization-prepared rapid gradient-echo sequence was used to acquire a series of 188 contiguous sagittal high resolution anatomical images with the following parameters: repetition time = 2000 ms, echo time = 2.26 ms, inversion time = 900 ms, flip angle = 9, matrix = 256×224, field of view = 256 mm×224 mm, slice thickness = 1 mm, which resulted in an isotropic voxel of 1 mm×1 mm×1 mm.

### MR data preprocessing

Structural images were preprocessed and analyzed using the Statistical Parametric Mapping software (SPM8; http://www.fil.ion.ucl.ac.uk/spm/software/spm8). The structural MR images were segmented into gray matter (GM), white matter and cerebrospinal fluid. GM images were subsequently spatially normalized into a Montreal Neurological Institute (MNI) space (http://www.mni.mcgill.ca/) with a resolution of 1.5 mm×1.5 mm×1.5 mm using the diffeomorphic anatomical registration through exponentiated lie algebra (DARTEL) method (Ashburner, 2007). GMV of each voxel was obtained by multiplying the GM concentration map by the non-linear determinants derived during spatial normalization. Here, the regional GMV represents normalized GMV after removing the effect of variance in individual brain sizes. Finally, the GMV images were smoothed with a full width at half maximum (FWHM) kernel of 8 mm. After spatial preprocessing, the normalized, modulated, and smoothed GMV maps were used for analysis of hippocampal volume and structural covariance.

### Voxel-wise GMV comparisons in hippocampus

**Figure 1:**
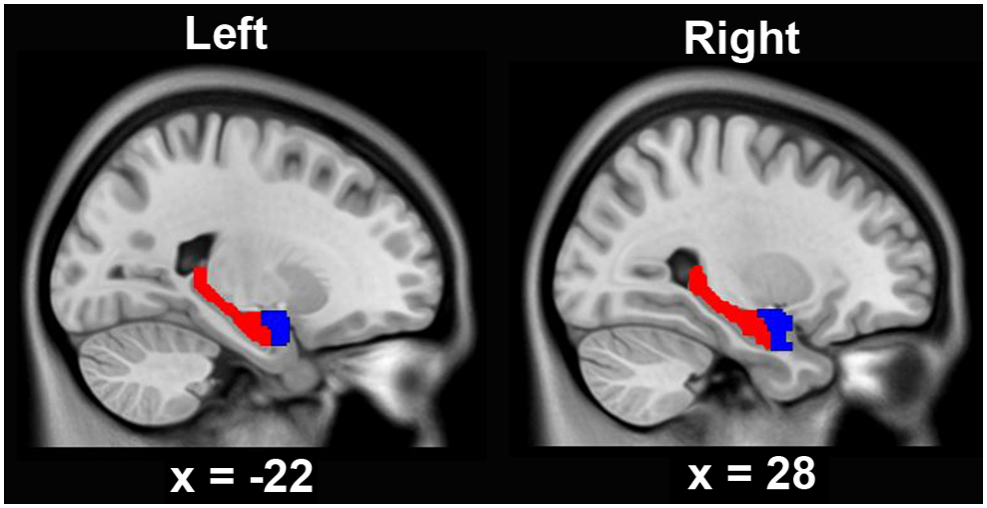
Combined hippocampal and amygdala mask on sagittal planes. Inter-group comparison of gray matter volume is performed within this mask. Left, left hemisphere; right, right hemisphere. The below numbers are the Montreal Neurological Institute coordinates on x axis.

The bilateral hippocampi and amygdalae were extracted using the Anatomical Automatic Labeling (AAL) atlas (www.cyceron.fr/freeware/); and they were combined and used as the mask during inter-group comparisons (**Figure 1**). Inter-group differences in GMV were voxel-wise compared within the mask using a two-sample *t*-test. A false discovery rate (FDR) method was used to correct for multiple comparisons, and a corrected threshold of *p*<0.05 as well as a minimum of 100 contiguous voxels was considered statistically significant.

Clusters with altered hippocampal GMV in PTSD patients were defined as regions of interest (ROIs). The GMVs in the hippocampal ROIs were extracted and the inter-group differences in these ROIs were further compared using a two-sample *t*-test, and the Cohen’s d was used to represent the effect size of the comparison. Considered that the anxiety and depression might confound the result, the one-way Analyses of Covariance (ANCOVA) controlling for HAMD and HAMA score (both separately and concurrently) were further performed to compare the inter-group differences of GMV in the hippocampal ROIs. A partial correlation analysis controlling for age effect was used to test correlations between GMV of the hippocampal ROIs and the PTSD symptom severity (the total and subtype scores of CAPS). Significance level was set at *p*<0.05.

### Structural covariance analysis

Voxel-wise GMV covariance quantifies the extent to which GMVs covary between different brain regions across participants. Here, the seed regions were defined as hippocampal ROIs that exhibited significant inter-group differences in GMV. The bilateral amygdalae were extracted with AAL atlas and were used as the mask. The GMV covariance between each seed region and voxels within ipsilateral amygdalar mask was obtained by calculating correlation coefficients between the GMV of the seed region and those of the ipsilateral amygdala voxels across all subjects of each group. Significance level was set at *p*<0.05, FDR correction for multiple comparisons, and with a minimum of 100 contiguous voxels.

A one-way ANCOVA was used to detect the inter-group differences in GMV covariance of the hippocampal ROIs with the ipsilateral amygdala (*p*<0.05, FDR corrected, cluster size >100 voxels). ROI-based analysis was also performed to validate the results of the voxel-wise analysis. Here, the amygdalar ROIs were defined as the amygdalar clusters exhibited significant inter-group difference in GMV covariance with the hippocampal ROIs. GMV correlations between the ipsilateral hippocampal and amygdalar ROIs were examined using the Pearson correlation analysis (*p*<0.05) in two groups, respectively.

### Statistical analysis for demographic and clinical variables

Kolmogorov-smirnov tests were performed to analyze the distribution of demographic and clinical variables. Differences between groups were analyzed with the Student’s *t* test in case of normally distributed variable (age) or with Mann-Whitney U test in case of non-normally distributed variables (HAMA and HAMD score) in SPSS11.0 (SPSS, Inc, Chiago.IL). Significance level was set at *P*<0.05.

## Results

### Demographic and clinical data

All subjects recruited in this study were male coalminers who had the same socioeconomic state and working environment. There were no significant differences in age and HAMA score between the two groups (*p*>0.05), but the HAMD score in PTSD patients was higher than that in controls (*p*<0.001). All non-PTSD subjects and 11 PTSD patients had the anxiety state (HAMA score ≥14). One non-PTSD subjects and 13 PTSD patients had the depression state (HAMD score ≥8). All the PTSD patients had no substance abuse before and after traumatic event. The demographic and clinical data are shown in **Table 1**.

**Table 1:** Demographic information of the PTSD patients and controls.

	PTSD (n = 14)	Controls (n = 25)	*statistics*	*p*
Age (years)	33.1±5.4	36.0±5.7	*t* = −1.497	0.143
HAMA	15.5(5–23)	15(14–20)	z = −0.400	0.689
HAMD	15(5–19)	2(0–10)	z = −5.059	<0.001
CAPS lifetime	87.6±14.1	-		
CAPS current	50.4±18.8	-		

HAMA and HAMD scores were non-normally distributed and showed as median (range). CAPS, Clinical-Administered PTSD scale; HAMA, Hamilton rating scale for anxiety; HAMD, Hamilton rating scale for Depression; PTSD, posttraumatic stress disorder.

### Reduced hippocampal volume

**Figure 2:**
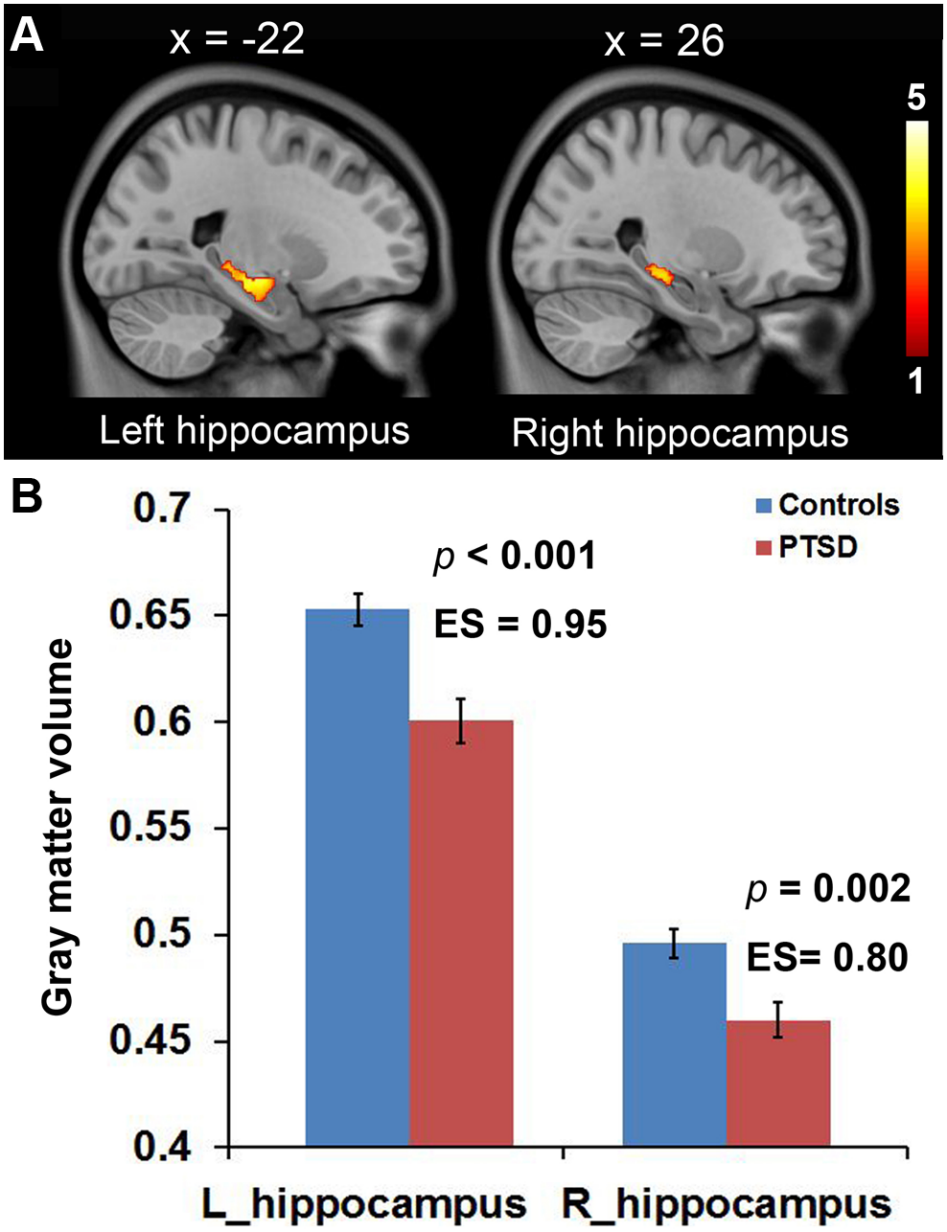
Reduced gray matter volumes of the bilateral hippocampi in PTSD patients. **A**, the color clusters exhibit reduced gray matter volume of bilateral hippocampi in PTSD patients compared with controls (*p*<0.05, FDR correction). The upper numbers are the Montreal Neurological Institute coordinates on x axis. The colorbar represents the statistical value from 1 to 5. **B**, bar graph shows the gray matter volumes in bilateral hippocampal ROIs significantly reduced in PTSD patients compared with controls exhibiting strong effect sizes. ES, effect size.

Coalminers with PTSD exhibited decreased GMV in the bilateral hippocampi compared to non-traumatized coalminers (**Figure 2A** **,** **Table 2**). ROI-based analysis showed that the volumes within the left and right hippocampal ROI decreased 7.96% (controls: 0.653±0.039, PTSD: 0.601±0.039; *p*<0.001, Cohen’s d = 0.95) and 7.26% (controls: 0.496±0.033, PTSD: 0.460±0.031; *p* = 0.002, Cohen’s d = 0.80) respectively in PTSD patients compared to controls (**Figure 2B**). The ANCOVA shows intergroup differences of GMV in the left hippocampal ROI was still significant after controlling the HAMD and HAMA score (both individually and concurrently). However, the intergroup difference of GMV in the right hippocampal ROI disappeared after controlling the HAMD scores or HAMD and HAMA scores concurrently (**Table 3**). There were no significant inter-group differences in GMVs of the bilateral amygdalae. In PTSD patients, there was no any statistical correlation between the hippocampal volume and the HAMA, HAMD, and CAPS score.

**Table 2:** Hippocampal clusters showed reduced gray matter volume in PTSD patients compared with controls.

Clusters	Voxels	MNI coordinates			*t* value	*p* a
		x	y	z		
Left hippocampus	731	–26	–15	–12	4.390	0.029
Right hippocampus	185	23	–24	–12	3.632	0.036

a FDR corrected at voxel level, *p*<0.05. MNI, Montreal Neurological Institute.

**Table 3:** Intergroup comparison of GMV in bilateral hippocampal ROIs.

	Left hippocampus		Right hippocampus	
Two-tailed *t*-test	*t* = 4.628	*P*<0.001	*t* = 3.413	*P* = 0.002
ANCOVA Controlling HAMA	*F* = 22.549	*P*<0.001	*F* = 10.617	*P* = 0.002
ANCOVA Controlling HAMD	*F* = 6.954	*P* = 0.012	*F* = 2.210	*P* = 0.146
ANCOVA Controlling HAMA and HAMD	*F* = 8.081	*P* = 0.007	*F* = 2.110	*P* = 0.155

ANCOVA, analysis of covariance; GMV, gray matter volume; HAMA, Hamilton rating scale for anxiety; HAMD, Hamilton rating scale for Depression.

### Structural covariance between the hippocampus and the ipsilateral amygdala

**Figure 3:**
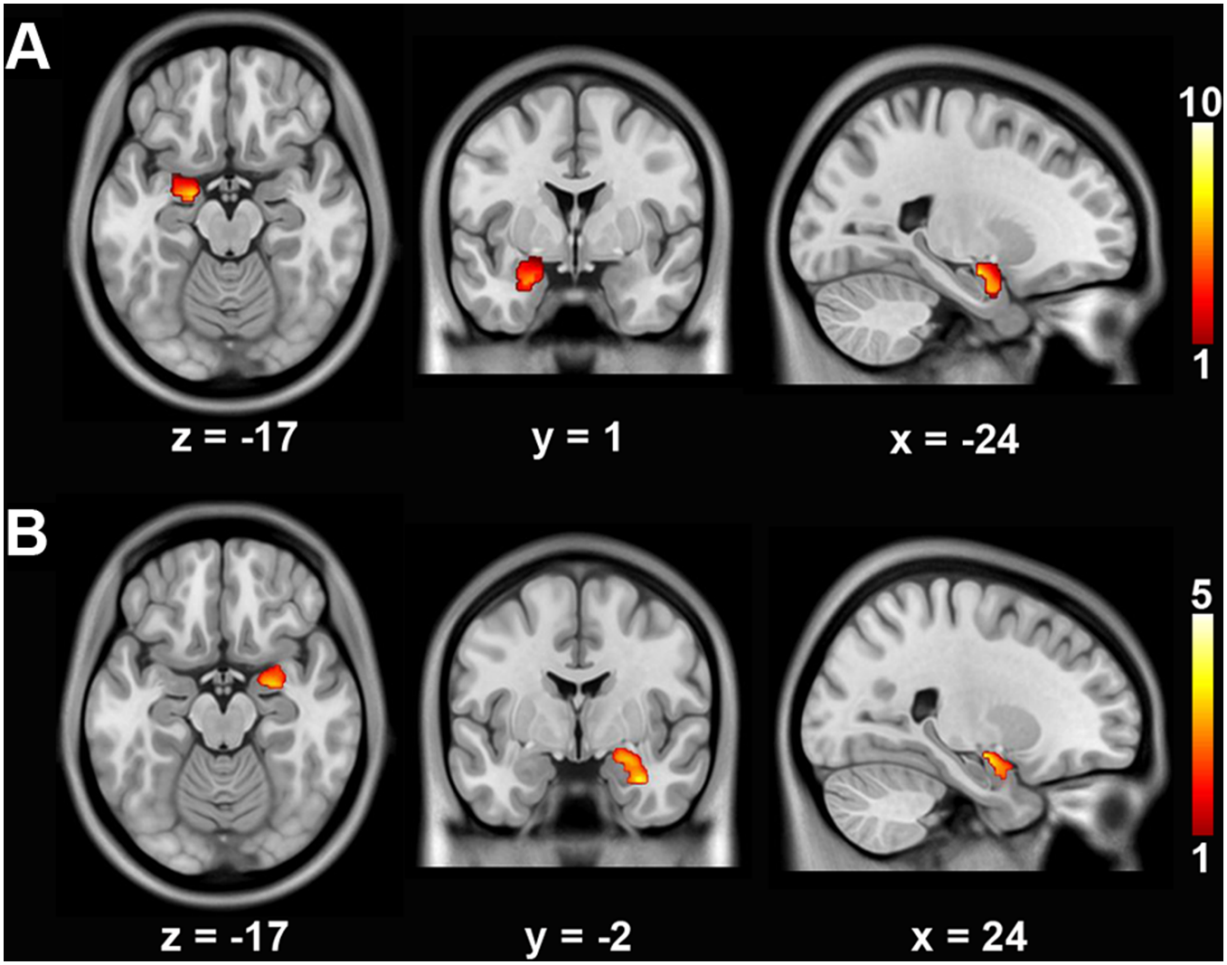
Gray matter volume (GMV) covariance of hippocampal ROIs with the ipsilateral amygdala in controls. The color regions represent the clusters within the left (**A**) and right (**B**) amygdala in which GMV significantly co-varied with the ipsilateral hippocampal ROI (*p*<0.05, FDR corrected). The colorbar represents the statistical value. The below numbers are the Montreal Neurological Institute coordinates.

Voxel-wise structural covariance analysis revealed that the GMV of the left hippocampal ROI significantly covaried with the left amygdala (MNI coordinate: –20, –9, –17; voxels: 478; *t* = 9.08, *p*<0.001, FDR corrected), and that the GMV of the right hippocampal ROI significantly covaried with the right amygdala (MNI coordinate: 32, –4, –27; voxels: 452; *t* = 4.10, *p* = 0.012, FDR corrected) in controls (**Figure 3**). However, there was no statistically significant GMV covariance between hippocampal ROIs and the ipsilateral amygdala in PTSD patients.

**Figure 4:**
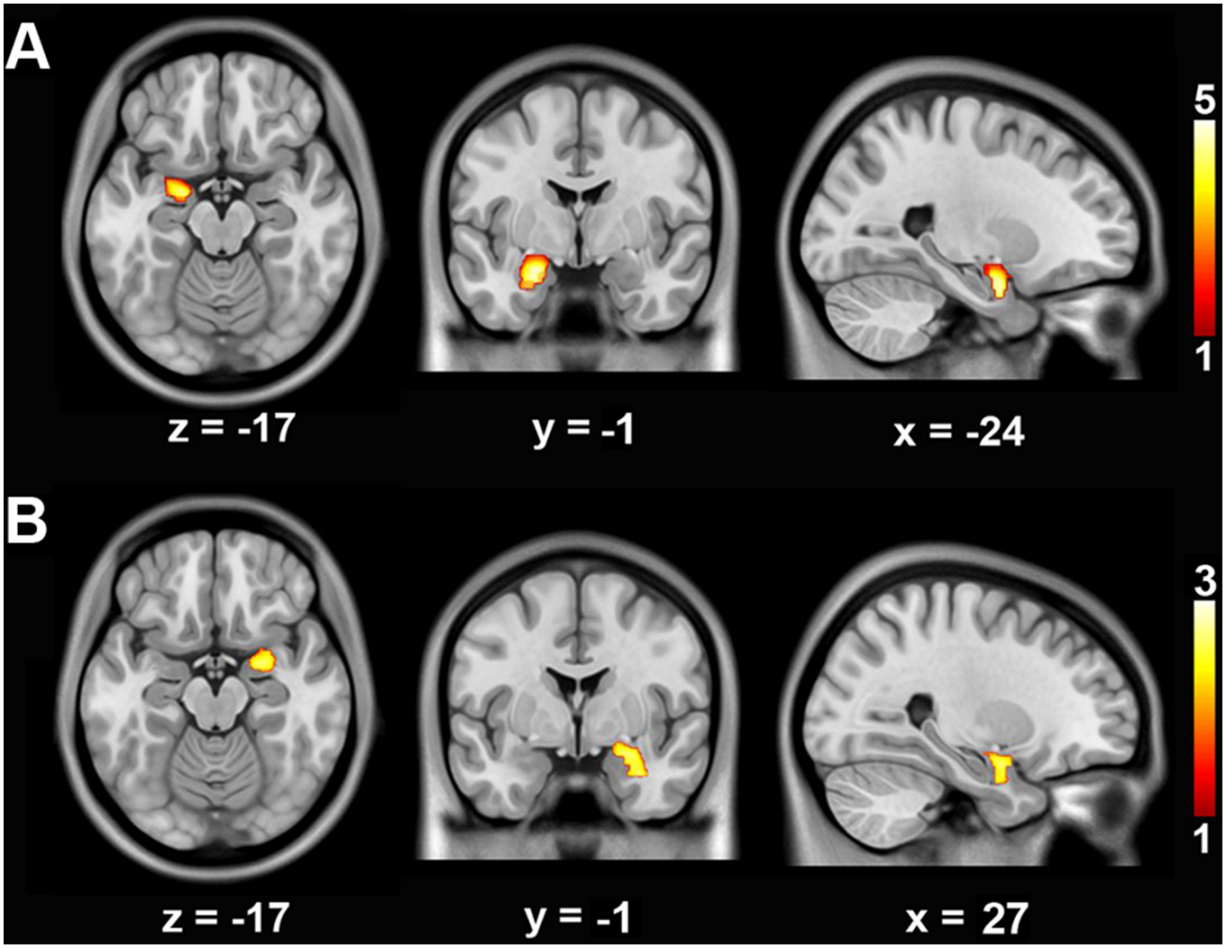
Inter-group differences in gray matter volume (GMV) covariance of hippocampal ROIs with the ipsilateral amygdala. The color regions represent the clusters within the left (**A**) and right (**B**) amygdala in which GMV covariance with the ipsilateral hippocampal ROI significantly decrease in PTSD patients compared with controls (*p*<0.05, FDR corrected). The colorbar represents the statistical value. The below numbers are the Montreal Neurological Institute coordinates.

**Figure 5:**
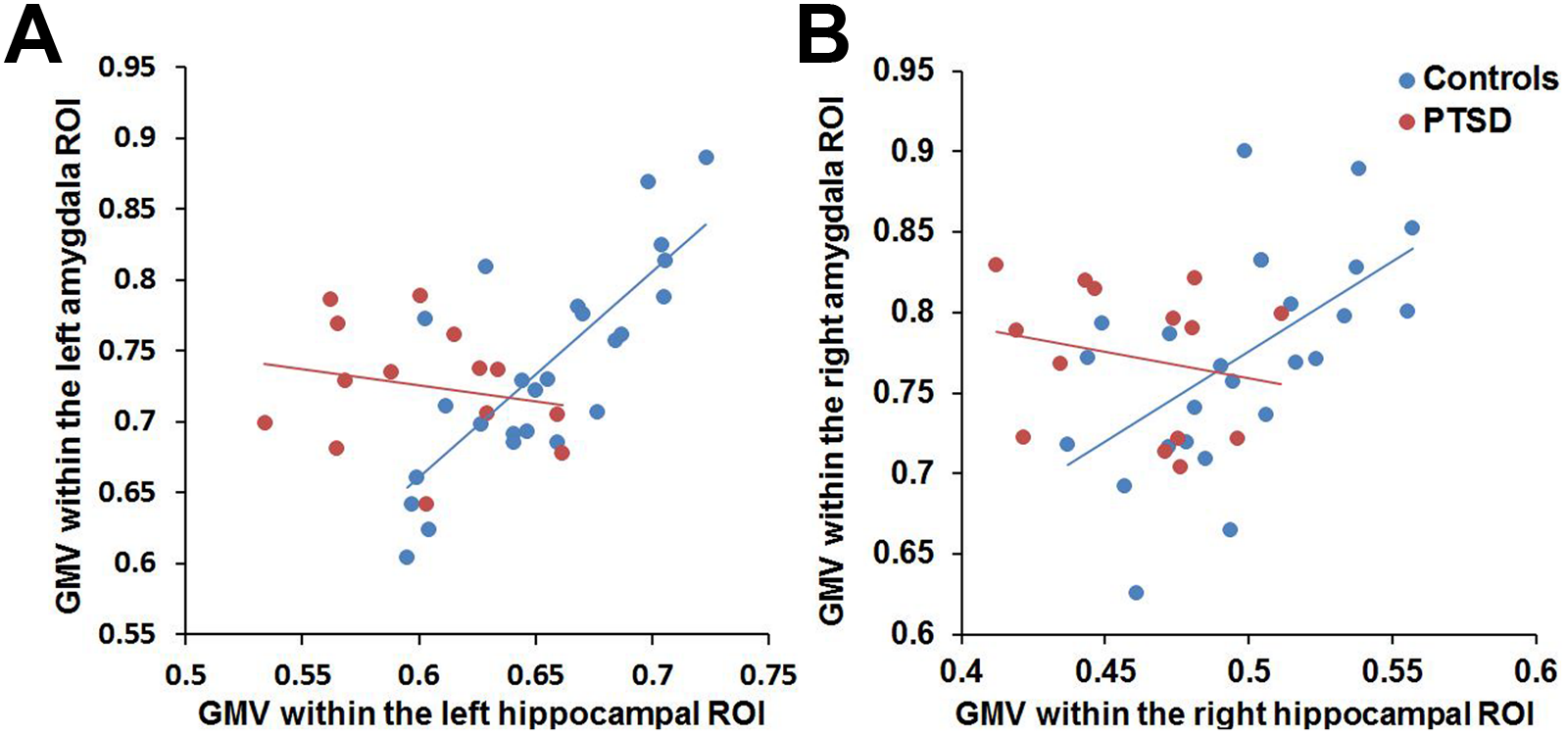
ROI-based correlation analyses of gray matter volumes between the bilateral hippocampal and amygdala ROIs. ROI-based correlation analyses revealed that decreased structural covariance between the left hippocampal ROI and the left amygdala ROI (**A**), and between the right hippocampal ROI and the right amygdala ROI (**B**) in PTSD patients were attributed to a strong positive correlation in controls (left side: *r* = 0.776, *p*<0.001; right side: *r* = 0.568, *p* = 0.003) and no significant correlation in PTSD patients (left side: *r = –*0.205, *p* = 0.483; right side: *r = *–0.219, *p* = 0.453).

Intergroup comparison of hippocampal structural covariance revealed a significantly reduced GMV covariance between the left hippocampal ROI and the left amygdala cluster (MNI coordinate: –21, –3, –21; voxels: 450; *t* = 4.87, *p*<0.001, FDR corrected), and between the right hippocampal ROI and the right amygdala cluster (MNI coordinate: 33, –3, –29; voxels: 374; *t* = 2.74, *p* = 0.042, FDR corrected) in the PTSD patients compared with controls (**Figure 4**). ROI-based correlation analysis showed strong positive correlations between the GMVs in the left hippocampus and the left amygdala (*r* = 0.776, *p*<0.001) as well as between the GMVs in the right hippocampus and the right amygdala (*r* = 0.568, *p* = 0.003) in healthy controls, while these GMV covariance disappeared in PTSD patients (left side: *r* = –0.205, *p* = 0.483; right side: *r* = –0.219, *p* = 0.453) (**Figure 5**).

## Discussion

In the present study, we investigated the alterations of the hippocampal and amygdalar volume and structural covariance between the hippocampus and the amygdala in coal mine gas explosion-related PTSD patients. We found that coalminers with PTSD had a significantly decreased GMV of hippocampus and decreased GMV covariance between the hippocampus and ipsilateral amygdala compared to non-traumatized coalminers, which may be associated with the dysfunction of emotional memory processing in PTSD patients.

### Reduced hippocampal volume in PTSD patients

Reduced volumes in bilateral hippocampi were revealed in coalminers with PTSD, especially in the left hippocampus, which is consistent with previous meta-analyses that reported a close association between the hippocampal volume and PTSD, although causal relationships between them remain unclear [6], [19], [20]. A study found that combat-exposed severe PTSD patients and their twins both had significantly smaller hippocampal volumes than non-PTSD twin pairs, suggesting that small hippocampal volume may be a risk factor for developing PTSD following trauma [39]. On the contrary, other studies demonstrated that the smaller hippocampal volume was a consequence of PTSD. Studies of animal model confirmed that traumatic experience could lead to reduced hippocampal volume [40], [41]. The reduction in hippocampal volume can be explained by the increased release of glucocorticoid under trauma exposure, which can affect hippocampal structure by causing atrophy of dendritic processes, neuronal loss, and decreased levels of brain-derived neurotrophic factor [42]. A longitudinal study on PTSD also demonstrated that stress could impair the human hippocampus [43]. The hippocampal volume loss is demonstrated negatively associated with the duration of PTSD [10]. Taken together, these findings might imply that small hippocampal volume predisposes to PTSD, and PTSD in turn leads to a secondary loss of hippocampal volume. Although it was difficult to determine whether smaller hippocampal volume was a preexisting risk factor or a consequence of the PTSD in this study, it is plausible to speculate that small hippocampal volume was associated with chronic severe PTSD following coal mine gas explosion.

In present study, the extent with decreased GMV in the left hippocampus was larger than that in the right hippocampus in coalminers with PTSD. The intergroup difference of the left hippocampal GMV was still significant after controlling the depression and/or anxiety, while not survive for the right hippocampus. Consistent with our finding, a meta-analysis found significant gray matter reduction of the left hippocampus in the PTSD patients compared with individuals exposed to trauma without PTSD, but no significant effect was found in the right hippocampus [22]. A study has shown that the left hippocampus was more vulnerable than the right one in the right-handed PTSD patients [44]. The right hippocampus appears particularly involved in memory for locations, whereas the left hippocampus is more involved in context-dependent episodic or autobiographical memory [45]. Because re-experiencing is one of the most important symptoms of PTSD, the memory about the traumatic events seems to influence more the left than the right hippocampus. However, other studies also found greater reductions in the right hippocampus in PTSD patients [46], [47]. The laterality of the hippocampal volume reduce should be further confirmed in PTSD patients with large sample sizes.

In this study, no significant correlation was revealed between bilateral hippocampal volume and the CAPS scores, which is inconsistent with previous findings of the correlation between hippocampal volume and the symptom severities of PTSD. Several studies found inversely correlations between hippocampal volume and the measures of symptom severity [38], [44], [48]–[51]. However, other studies had also shown no associations between bilateral hippocampal volume and the CAPS scores [7], [9], [15], [18]. Multiple variables could attribute to these inconsistent results, including sample size, age and gender, trauma type and duration, analytic methods [19], [23], and comorbid disorders [52]. The possible explanation for our result might be that the small hippocampus was a preexisting risk factor for PTSD and was only a trait marker but not state marker of PTSD. Other factors such as the small sample size and long disease course might also contribute to this negative result. However, further study with large sample size should be performed to clarify whether definitive correlation exists between the symptom severity and hippocampal volume in PTSD.

### Structural covariance between hippocampus and ipsilateral amygdala

We found that the hippocampus had a deceased GMV covariance with the ipsilateral amygdala in coalminers with PTSD compared with non-traumatized coalminers. ROI-based correlation analysis revealed that this abnormal GMV covariance between the two regions could be attributable to the disrupted positive association in coalminers with PTSD

Brain regions that covary with each other are often parts of a system that subserves particular behavioral or cognitive function [36]. In the healthy adults, the hippocampal volume covaries strongly with those of brain regions involved in the memory system, including the amygdala and parahippocampal, perirhinal, entorhinal and orbitalfrontal cortices [34]. Our study also revealed that the hippocampus strongly positively covaried with the ipsilateral amygdala in non-traumatized coalminers. However, the structural covariance between hippocampus and ipsilateral amygdala was disrupted in PTSD group. One possible explanation might be that there are extensive anatomical interconnections between the hippocampus and amygdala [32], [33]. Animal studies demonstrated that stress elicits hypertrophy of amygdala [53], but atrophy in hippocampus [54]. The structural synchronization between hippocampus and ipsilateral amygdala might be destroyed in PTSD. Another possible explanation for decreased structural covariance between hippocampus and ipsilateral amygdala was associated to the contrary functional activities of the two regions. Functional imaging studies on PTSD revealed increased activation in amygdala and decreased activation in hippocampus during traumatic script-driven imaginary task [55]. Growing dominance of amygdalar activity over the hippocampus during and even after chronic stress may contribute to the enhanced emotional symptoms seen in stress-related psychiatric disorders [56]. Amygdala may mediate certain stress effects on hippocampus [57]. The destroyed structural covariance between hippocampus and ipsilateral amygdala might be secondary to the long term non-synchronized functional activity of two regions. Overall,decreased GMV covariance between the hippocampus and the ipsilateral amygdala might be associated with abnormal processing of trauma-related memory such as re-experiencing, which was one of the most important symptoms in PTSD patients.

### Limitations

Several limitations should be considered in this study. First, the relatively small sample size may impede for detecting significant effects because of the lack of power. However, high homogeneity of the PTSD patients who suffering from coal mine gas explosion might compensate this shortage to some extent. Second, the specificity of trauma event was coal mine gas explosion. Although patients with visible brain lesions have been excluded, we cannot exclude the possibility of the subtle damage caused by poisoning. Third, although the comorbid depression might affect the our results, ANCOVA result showed that intergroup GMV difference was still significant in the left hippocampal after controlling the HAMD, indicating close association between decreased left hippocampal GMV and PTSD. Finally, only the coalminers without trauma exposure were enrolled as controls. Future studies should also include non-PTSD controls with trauma exposure.

## Conclusion

To our knowledge, this is the first study to investigate the structural covariance between the hippocampus and amygdala in PTSD. We found that bilateral hippocampal GMVs decreased in coal mine gas explosion related PTSD patients, especially the left hippocampus; and the hippocampal regions exhibiting reduced GMV had decreased structural covariance with the ipsilateral amygdala, which may be associated with the symptom of the PTSD.

## References

[1] Rauch SL, Shin LM, Phelps EA (2006) Neurocircuitry models of posttraumatic stress disorder and extinction: human neuroimaging research–past, present, and future. Biological Psychiatry 60: 376–382. 16919525 10.1016/j.biopsych.2006.06.004

[2] Liberzon I, Sripada CS (2008) The functional neuroanatomy of PTSD: a critical review. Prog Brain Res 167: 151–169. 18037013 10.1016/S0079-6123(07)67011-3

[3] Mitra R, Jadhav S, McEwen BS, Vyas A, Chattarji S (2005) Stress duration modulates the spatiotemporal patterns of spine formation in the basolateral amygdala. Proceedings of the National Academy of Sciences of the United States of America 102: 9371–9376. 15967994 10.1073/pnas.0504011102 PMC1166638

[4] Bannerman DM, Rawlins JN, McHugh SB, Deacon RM, Yee BK, et al (2004) Regional dissociations within the hippocampus–memory and anxiety. Neuroscience and biobehavioral reviews 28: 273–283. 15225971 10.1016/j.neubiorev.2004.03.004

[5] Fanselow MS, Dong HW (2010) Are the dorsal and ventral hippocampus functionally distinct structures? Neuron 65: 7–19. 20152109 10.1016/j.neuron.2009.11.031 PMC2822727

[6] Karl A, Schaefer M, Malta LS, Dorfel D, Rohleder N, et al (2006) A meta-analysis of structural brain abnormalities in PTSD. Neuroscience and biobehavioral reviews 30: 1004–1031. 16730374 10.1016/j.neubiorev.2006.03.004

[7] Bonne O, Vythilingam M, Inagaki M, Wood S, Neumeister A, et al (2008) Reduced posterior hippocampal volume in posttraumatic stress disorder. Journal of Clinical Psychiatry 69: 1087–1091. 18572983 10.4088/jcp.v69n0707 PMC2684983

[8] Emdad R, Bonekamp D, Sondergaard HP, Bjorklund T, Agartz I, et al (2006) Morphometric and psychometric comparisons between non-substance-abusing patients with posttraumatic stress disorder and normal controls. Psychotherapy and psychosomatics 75: 122–132. 16508348 10.1159/000090897

[9] Chen S, Xia W, Li L, Liu J, He Z, et al (2006) Gray matter density reduction in the insula in fire survivors with posttraumatic stress disorder: a voxel-based morphometric study. Psychiatry Research 146: 65–72. 16371250 10.1016/j.pscychresns.2005.09.006

[10] Felmingham K, Williams LM, Whitford TJ, Falconer E, Kemp AH, et al (2009) Duration of posttraumatic stress disorder predicts hippocampal grey matter loss. Neuroreport 20: 1402–1406. 19794316 10.1097/WNR.0b013e3283300fbc

[11] Thomaes K, Dorrepaal E, Draijer N, de Ruiter MB, van Balkom AJ, et al (2010) Reduced anterior cingulate and orbitofrontal volumes in child abuse-related complex PTSD. Journal of Clinical Psychiatry 71: 1636–1644. 20673548 10.4088/JCP.08m04754blu

[12] Wang Z, Neylan TC, Mueller SG, Lenoci M, Truran D, et al (2010) Magnetic resonance imaging of hippocampal subfields in posttraumatic stress disorder. Archives General Psychiatry 67: 296–303. 10.1001/archgenpsychiatry.2009.205 PMC2848481 20194830

[13] Shin LM, Shin PS, Heckers S, Krangel TS, Macklin ML, et al (2004) Hippocampal function in posttraumatic stress disorder. Hippocampus 14: 292–300. 15132428 10.1002/hipo.10183

[14] Yehuda R, Golier JA, Tischler L, Harvey PD, Newmark R, et al (2007) Hippocampal volume in aging combat veterans with and without post-traumatic stress disorder: relation to risk and resilience factors. Journal of Psychiatric Research 41: 435–445. 16445942 10.1016/j.jpsychires.2005.12.002

[15] Bonne O, Brandes D, Gilboa A, Gomori JM, Shenton ME, et al (2001) Longitudinal MRI study of hippocampal volume in trauma survivors with PTSD. The American Journal of Psychiatry 158: 1248–1251. 11481158 10.1176/appi.ajp.158.8.1248 PMC2819102

[16] Fennema-Notestine C, Stein MB, Kennedy CM, Archibald SL, Jernigan TL (2002) Brain morphometry in female victims of intimate partner violence with and without posttraumatic stress disorder. Biological Psychiatry 52: 1089–1101. 12460692 10.1016/s0006-3223(02)01413-0

[17] Golier JA, Yehuda R, De Santi S, Segal S, Dolan S, et al (2005) Absence of hippocampal volume differences in survivors of the Nazi Holocaust with and without posttraumatic stress disorder. Psychiatry Research 139: 53–64. 15939577 10.1016/j.pscychresns.2005.02.007

[18] Pederson CL, Maurer SH, Kaminski PL, Zander KA, Peters CM, et al (2004) Hippocampal volume and memory performance in a community-based sample of women with posttraumatic stress disorder secondary to child abuse. Journal of traumatic stress 17: 37–40. 15027791 10.1023/B:JOTS.0000014674.84517.46

[19] Smith ME (2005) Bilateral hippocampal volume reduction in adults with post-traumatic stress disorder: a meta-analysis of structural MRI studies. Hippocampus 15: 798–807. 15988763 10.1002/hipo.20102

[20] Kitayama N, Vaccarino V, Kutner M, Weiss P, Bremner JD (2005) Magnetic resonance imaging (MRI) measurement of hippocampal volume in posttraumatic stress disorder: a meta-analysis. Journal of affective disorders 88: 79–86. 16033700 10.1016/j.jad.2005.05.014

[21] Woon FL, Sood S, Hedges DW (2010) Hippocampal volume deficits associated with exposure to psychological trauma and posttraumatic stress disorder in adults: a meta-analysis. Progress in Neuro-psychopharmacology & Biological Psychiatry 34: 1181–1188. 20600466 10.1016/j.pnpbp.2010.06.016

[22] Kuhn S, Gallinat J (2013) Gray matter correlates of posttraumatic stress disorder: a quantitative meta-analysis. Biological Psychiatry 73: 70–74. 22840760 10.1016/j.biopsych.2012.06.029

[23] Nemeroff CB, Bremner JD, Foa EB, Mayberg HS, North CS, et al (2006) Posttraumatic stress disorder: a state-of-the-science review. Journal of Psychiatric Research 40: 1–21. 16242154 10.1016/j.jpsychires.2005.07.005

[24] Kuo JR, Kaloupek DG, Woodward SH (2012) Amygdala volume in combat-exposed veterans with and without posttraumatic stress disorder: a cross-sectional study. Arch Gen Psychiatry 69: 1080–1086. 23026958 10.1001/archgenpsychiatry.2012.73

[25] Rogers MA, Yamasue H, Abe O, Yamada H, Ohtani T, et al (2009) Smaller amygdala volume and reduced anterior cingulate gray matter density associated with history of post-traumatic stress disorder. Psychiatry Res 174: 210–216. 19914045 10.1016/j.pscychresns.2009.06.001

[26] Morey RA, Gold AL, LaBar KS, Beall SK, Brown VM, et al (2012) Amygdala volume changes in posttraumatic stress disorder in a large case-controlled veterans group. Arch Gen Psychiatry 69: 1169–1178. 23117638 10.1001/archgenpsychiatry.2012.50 PMC3647246

[27] Woon FL, Hedges DW (2008) Hippocampal and amygdala volumes in children and adults with childhood maltreatment-related posttraumatic stress disorder: a meta-analysis. Hippocampus 18: 729–736. 18446827 10.1002/hipo.20437

[28] Woon FL, Hedges DW (2009) Amygdala volume in adults with posttraumatic stress disorder: a meta-analysis. J Neuropsychiatry Clin Neurosci 21: 5–12. 19359446 10.1176/jnp.2009.21.1.5

[29] Dolcos F, LaBar KS, Cabeza R (2004) Interaction between the amygdala and the medial temporal lobe memory system predicts better memory for emotional events. Neuron 42: 855–863. 15182723 10.1016/s0896-6273(04)00289-2

[30] Smith APR, Stephan KE, Rugg MD, Dolan RJ (2006) Task and Content Modulate Amygdala-Hippocampal Connectivity in Emotional Retrieval. Neuron 49: 631–638. 16476670 10.1016/j.neuron.2005.12.025

[31] Vaisvaser S, Lin T, Admon R, Podlipsky I, Greenman Y, et al. (2013) Neural traces of stress: cortisol related sustained enhancement of amygdala-hippocampal functional connectivity. Frontiers in Human Neuroscience 7. 10.3389/fnhum.2013.00313 PMC3701866 23847492

[32] Kishi T, Tsumori T, Yokota S, Yasui Y (2006) Topographical projection from the hippocampal formation to the amygdala: a combined anterograde and retrograde tracing study in the rat. J Comp Neurol 496: 349–368. 16566004 10.1002/cne.20919

[33] Pitkanen A, Pikkarainen M, Nurminen N, Ylinen A (2000) Reciprocal connections between the amygdala and the hippocampal formation, perirhinal cortex, and postrhinal cortex in rat. A review. Ann N Y Acad Sci 911: 369–391. 10911886 10.1111/j.1749-6632.2000.tb06738.x

[34] Bohbot VD, Lerch J, Thorndycraft B, Iaria G, Zijdenbos AP (2007) Gray matter differences correlate with spontaneous strategies in a human virtual navigation task. The Journal of neuroscience 27: 10078–10083. 17881514 10.1523/JNEUROSCI.1763-07.2007 PMC6672675

[35] Mechelli A, Friston KJ, Frackowiak RS, Price CJ (2005) Structural covariance in the human cortex. The Journal of neuroscience 25: 8303–8310. 16148238 10.1523/JNEUROSCI.0357-05.2005 PMC6725541

[36] Alexander-Bloch A, Giedd JN, Bullmore E (2013) Imaging structural co-variance between human brain regions. Nature reviews Neuroscience 14: 322–336. 23531697 10.1038/nrn3465 PMC4043276

[37] Wang HH, Zhang ZJ, Tan QR, Yin H, Chen YC, et al (2010) Psychopathological, biological, and neuroimaging characterization of posttraumatic stress disorder in survivors of a severe coalmining disaster in China. Journal of Psychiatric Research 44: 385–392. 19896142 10.1016/j.jpsychires.2009.10.001

[38] Zhang J, Tan Q, Yin H, Zhang X, Huan Y, et al (2011) Decreased gray matter volume in the left hippocampus and bilateral calcarine cortex in coal mine flood disaster survivors with recent onset PTSD. Psychiatry Research 192: 84–90. 21498053 10.1016/j.pscychresns.2010.09.001

[39] Gilbertson MW, Shenton ME, Ciszewski A, Kasai K, Lasko NB, et al (2002) Smaller hippocampal volume predicts pathologic vulnerability to psychological trauma. Nature neuroscience 5: 1242–1247. 12379862 10.1038/nn958 PMC2819093

[40] Golub Y, Kaltwasser SF, Mauch CP, Herrmann L, Schmidt U, et al (2011) Reduced hippocampus volume in the mouse model of Posttraumatic Stress Disorder. Journal of Psychiatric Research 45: 650–659. 21106206 10.1016/j.jpsychires.2010.10.014

[41] Liu W, Shu XJ, Chen FY, Zhu C, Sun XH, et al (2011) Tianeptine reverses stress-induced asymmetrical hippocampal volume and N-acetylaspartate loss in rats: an in vivo study. Psychiatry Research 194: 385–392. 22047727 10.1016/j.pscychresns.2011.02.007

[42] Uno H, Eisele S, Sakai A, Shelton S, Baker E, et al (1994) Neurotoxicity of glucocorticoids in the primate brain. Hormones and behavior 28: 336–348. 7729802 10.1006/hbeh.1994.1030

[43] Carrion VG, Weems CF, Reiss AL (2007) Stress predicts brain changes in children: a pilot longitudinal study on youth stress, posttraumatic stress disorder, and the hippocampus. Pediatrics 119: 509–516. 17332204 10.1542/peds.2006-2028

[44] Shu XJ, Xue L, Liu W, Chen FY, Zhu C, et al (2012) More vulnerability of left than right hippocampal damage in right-handed patients with post-traumatic stress disorder. Psychiatry Research 212: 237–244. 23149034 10.1016/j.pscychresns.2012.04.009

[45] Burgess N, Maguire EA, O’Keefe J (2002) The human hippocampus and spatial and episodic memory. Neuron 35: 625–641. 12194864 10.1016/s0896-6273(02)00830-9

[46] Pavic L, Gregurek R, Rados M, Brkljacic B, Brajkovic L, et al (2007) Smaller right hippocampus in war veterans with posttraumatic stress disorder. Psychiatry Research 154: 191–198. 17258898 10.1016/j.pscychresns.2006.08.005

[47] Wignall EL, Dickson JM, Vaughan P, Farrow TF, Wilkinson ID, et al (2004) Smaller hippocampal volume in patients with recent-onset posttraumatic stress disorder. Biological Psychiatry 56: 832–836. 15576059 10.1016/j.biopsych.2004.09.015

[48] Vythilingam M, Luckenbaugh DA, Lam T, Morgan CA 3rd, Lipschitz D, et al (2005) Smaller head of the hippocampus in Gulf War-related posttraumatic stress disorder. Psychiatry Research 139: 89–99. 15967648 10.1016/j.pscychresns.2005.04.003

[49] Bremner JD, Vythilingam M, Vermetten E, Southwick SM, McGlashan T, et al (2003) MRI and PET study of deficits in hippocampal structure and function in women with childhood sexual abuse and posttraumatic stress disorder. The American Journal of Psychiatry 160: 924–932. 12727697 10.1176/appi.ajp.160.5.924

[50] Villarreal G, Hamilton DA, Petropoulos H, Driscoll I, Rowland LM, et al (2002) Reduced hippocampal volume and total white matter volume in posttraumatic stress disorder. Biological Psychiatry 52: 119–125. 12114003 10.1016/s0006-3223(02)01359-8

[51] Lindauer RJ, Vlieger EJ, Jalink M, Olff M, Carlier IV, et al (2004) Smaller hippocampal volume in Dutch police officers with posttraumatic stress disorder. Biological Psychiatry 56: 356–363. 15336518 10.1016/j.biopsych.2004.05.021

[52] Sheline YI, Sanghavi M, Mintun MA, Gado MH (1999) Depression duration but not age predicts hippocampal volume loss in medically healthy women with recurrent major depression. The Journal of neuroscience 19: 5034–5043. 10366636 10.1523/JNEUROSCI.19-12-05034.1999 PMC6782668

[53] Vyas A, Pillai AG, Chattarji S (2004) Recovery after chronic stress fails to reverse amygdaloid neuronal hypertrophy and enhanced anxiety-like behavior. Neuroscience 128: 667–673. 15464275 10.1016/j.neuroscience.2004.07.013

[54] Luine V, Villegas M, Martinez C, McEwen BS (1994) Repeated stress causes reversible impairments of spatial memory performance. Brain Res 639: 167–170. 8180832 10.1016/0006-8993(94)91778-7

[55] Hughes KC, Shin LM (2011) Functional neuroimaging studies of post-traumatic stress disorder. Expert Rev Neurother 11: 275–285. 21306214 10.1586/ern.10.198 PMC3142267

[56] Ghosh S, Laxmi TR, Chattarji S (2013) Functional Connectivity from the Amygdala to the Hippocampus Grows Stronger after Stress. Journal of Neuroscience 33: 7234–7244. 23616532 10.1523/JNEUROSCI.0638-13.2013 PMC6619590

[57] Kim JJ, Lee HJ, Han JS, Packard MG (2001) Amygdala is critical for stress-induced modulation of hippocampal long-term potentiation and learning. J Neurosci 21: 5222–5228. 11438597 10.1523/JNEUROSCI.21-14-05222.2001 PMC6762855

